# Prognostic Significance of a Novel Histopathologic Risk Model Incorporating Modifications to the Worst Pattern of Invasion and Tumor Budding for Oral Squamous Cell Carcinoma

**DOI:** 10.1111/jop.70046

**Published:** 2025-08-19

**Authors:** Everton Freitas de Morais, Lívia Maris Ribeiro Paranaiba Dias, Ana Lúcia Carrinho Ayroza Rangel, Ricardo D. Coletta

**Affiliations:** ^1^ Graduate Program in Oral Biology, Piracicaba Dental School University of Campinas Piracicaba São Paulo Brazil; ^2^ Department of Oral Diagnosis, Piracicaba Dental School University of Campinas Piracicaba São Paulo Brazil; ^3^ Department of Pathology and Parasitology, Institute of Biomedical Sciences Federal University of Alfenas Alfenas Minas Gerais Brazil; ^4^ Center of Biological Sciences and of the Health, School of Dentistry State University of Western Paraná Cascavel Paraná Brazil

**Keywords:** histopathologic risk model, oral cancer, prognosis, tumor budding, worst pattern of invasion

## Abstract

**Background:**

Prognostic stratification to predict patient outcomes and guide treatment decisions is urgently needed to improve clinical management of oral squamous cell carcinoma (OSCC). This study evaluated the prognostic performance of a novel histopathologic risk model that integrates modifications to the worst pattern of invasion (WPOI) system and tumor budding (TB) classification for OSCC, focusing on its effectiveness in predicting recurrence and survival.

**Methods:**

In a retrospective study of 193 OSCC cases, the modifications incorporated to the WPOI and TB were initially compared to conventional assessments. Next, the effectiveness of the novel histopathologic risk model was assessed. Univariate and multivariate survival analyses were performed using Cox proportional hazards models. The performance of the classifiers was evaluated by applying the receiver operating characteristic (ROC) analysis, calculating the area under the curve (AUC).

**Results:**

Classical WPOI was significantly and independently associated with shortened cancer‐specific survival (HR = 2.0, 95% CI = 1.26–3.25, *p* = 0.003); whereas classical TB was associated with poor disease‐free survival (HR = 2.17, 95% CI = 1.25–3.74, *p* = 0.005). In contrast, the modified WPOI and TB showed no significant associations with patient outcomes. The novel histopathologic risk model, which categorized 68.4% of cases as intermediate risk, showed limited ability to predict OSCC outcomes.

**Conclusions:**

The findings of this study show the superior prognostic value of the classical WPOI and TB over their modified counterparts; the potential of the novel histopathologic risk was not validated in this cohort including OSCCs at all clinical stages.

## 1 Introduction

Oral squamous cell carcinoma (OSCC) is the most common malignant neoplasm of the head and neck, with a challenging prognosis and a 5‐year survival rate of approximately 50% [1]. The clinical management of OSCC is primarily guided by clinical stage and a few selected histopathological features [2, 3], which support decision‐making as an expanding range of treatment options becomes available. Although the latest edition of TNM clinical staging has incorporated histopathological markers such as depth of invasion (DOI) and extranodal extension (ENE) [4], bringing prognostic accuracy, gaps remain regarding the clinical applicability of several histopathological markers [3].

The worst pattern of invasion (WPOI) and tumor budding (TB) are promising prognostic markers. WPOI is associated with adverse clinicopathological parameters, including lymphovascular invasion, ENE, and positive surgical margins, leading to poor survival and high rates of locoregional recurrence [5, 6]. Similarly, TB, defined as tumor clusters with < 5 cells which reflect cellular dissociation, was highly linked to recurrence and mortality [7, 8]. However, classical WPOI classification suffers from overlapping criteria, while TB assessment lacks standardization [9]. Chang et al. [9] proposed recently a novel histopathological risk model integrating modifications to both WPOI and TB. The modified WPOI consolidated the five tiers into three by merging WPOI‐1 (pushing border growth) and WPOI‐2 (finger‐like growth) into a single category, and grouping large (> 15 cells, WOPI‐3) and small (< 15 cells, WPOI‐4) tumor islands together. The modified TB scoring incorporated isolated cells and was divided into three levels: 0 (absence of buds), 1 (presence of 1–4 buds), and 2 (≥ 5 buds or presence of isolated cells). This study aimed to evaluate the prognostic performance of the modified WPOI and TB, and of the recently described histopathologic risk model in patients with OSCC at all clinical stages.

## 2 Materials and Methods

The study explored a cohort composed of 193 OSCC cases, with the complete description of the demographic, clinical, and pathological features having been previously reported elsewhere [10]. The main clinicopathological characteristics of the patients are depicted in Data S1. Histological slides stained with hematoxylin and eosin from the surgical resections were retrieved for evaluation. The WPOI was assessed following the original description by Brandwein‐Gensler et al. [11], and TB was evaluated and classified according to the criteria reported by Almangush et al. [7]. The modified WPOI and modified TB were individually assessed on the same slides and later categorized in risk scores (low, intermediate and high risk) according to the original study of Chang et al. [9]. The histopathological parameters of analysis are described and illustrated in Data S2. Two pathologists, blinded to clinicopathological features and previously calibrated (inter‐observer Cohen's *κ* agreement rates of 0.95 for modified WPOI, 0.93 for modified TB, 0.94 for WPOI, and 0.89 for TB), independently scored the histopathological parameters.

Cancer‐specific survival and disease‐free survival were assessed using univariable and multivariable Cox regression models, and survival curves were constructed according to the Kaplan–Meier method and compared by the log‐rank test. A receiver operating characteristic (ROC) curve with area under the curve (AUC) was applied to compare the discriminatory ability of the systems. This study was approved by the Human Research Ethics Committee of the School of Dentistry, University of Campinas (CAAE: 55927322.0.0000.5418).

## 3 Results

The analysis of 193 OSCCs demonstrated distinct distributions for classical and modified classifications of WPOI and TB (Data S3). According to the classical WPOI, most cases were classified as WPOI‐3 (large tumor islands, 53.9%) or WPOI‐4 (small tumor islands, 33.1%), and the modified WPOI classified 91.2% of cases as pattern 1 (presence of tumor islands of any size). TB assessment after the original classification system showed a quite similar distribution, whereas the modified TB model classified 50.3% of cases as score 2 (≥ 5 buds or presence of isolated cells). Applying the novel histopathological risk model, 55 (28.5%) cases were classified as low risk, 132 (68.4%) as intermediate risk, and 6 (3.1%) as high risk (Data S3).

**FIGURE 1:**
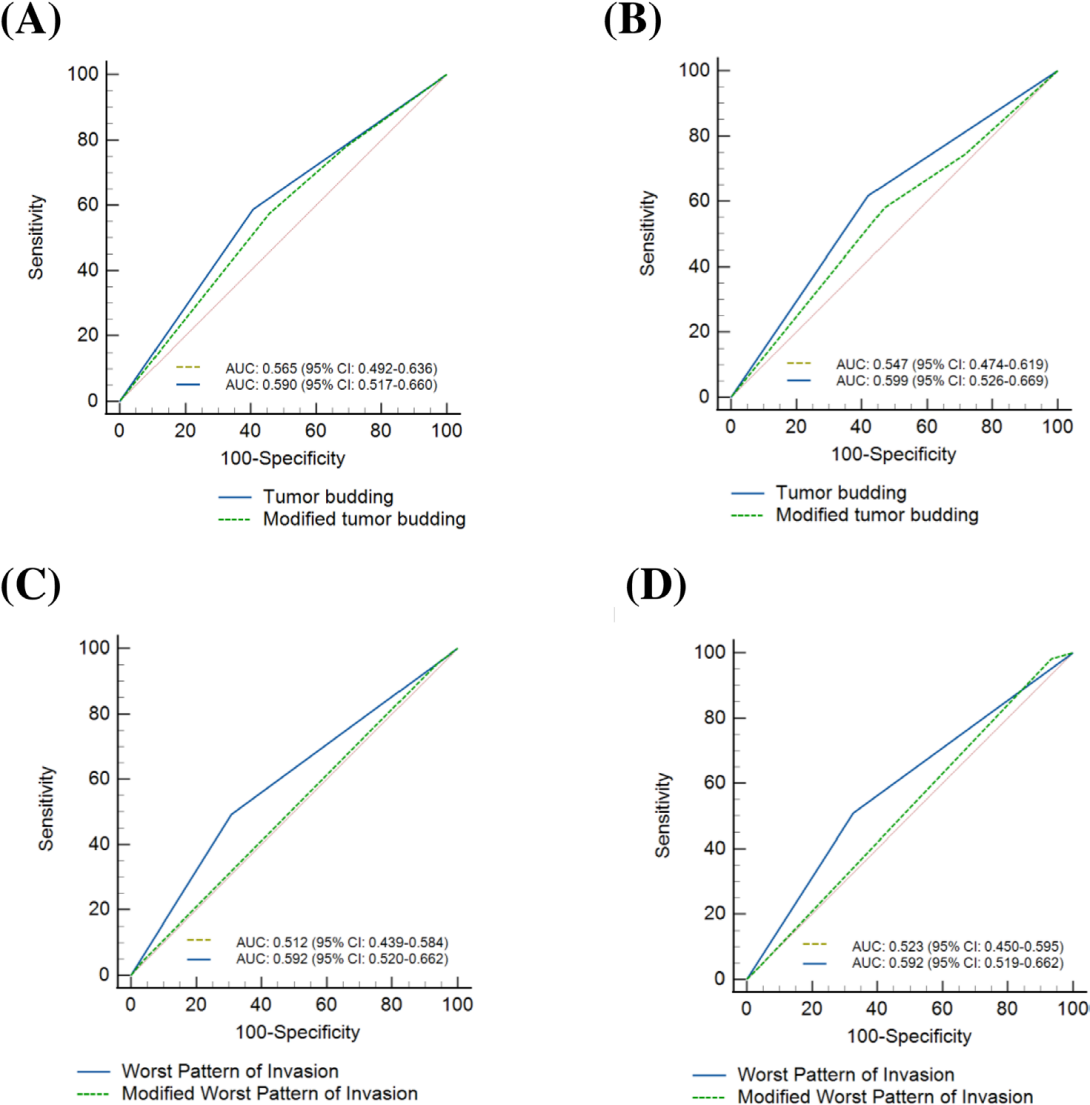
Receiver operating characteristic (ROC) analysis for prognostic value of classical and modified worst pattern of invasion (WPOI) and tumor budding (TB) in oral squamous cell carcinoma. (A) ROC curve for TB predicting mortality, (B) ROC curve for TB predicting recurrence, (C) ROC curve for WPOI predicting mortality, and (D) ROC curve for WPOI predicting recurrence.

WPOI and TB, but not modified WPOI or modified TB, were significantly associated with cancer‐specific survival and disease‐free survival in univariate analysis (Table 1). However, multivariate analyses, adjusted by the clinicopathological features, identified WPOI as an independent predictor of cancer‐specific survival (HR = 2.02, 95% CI = 1.26–3.25, *p* = 0.003) and TB as an independent predictor of disease‐free survival (HR = 2.17, 95% CI = 1.25–3.74, *p* = 0.005) (Table 2). To further characterize the systems (classical and modified), the AUC values from ROC curves were compared. As depicted in Figure 1, the WPOI and TB showed superior discrimination, with higher AUC values, compared to modified WPOI and modified TB.

**TABLE 1:** Univariate analysis for cancer‐specific survival and disease‐free survival of 193 patients with oral squamous cell carcinoma.

	Cancer‐specific survival		Disease‐free survival	
	HR (95% CI)	*p*	HR (95% CI)	*p*
Age (years)				
< 58 years	1		1	
≥ 58 years	1.43 (0.89–2.28)	0.13	0.95 (0.56–1.61)	0.85
Sex				
Male	1		1	
Female	0.80 (0.45–1.45)	0.47	0.84 (0.43–1.62)	0.60
Smoking				
Never‐smoker	1		1	
Smoker or Former‐smoker	1.08 (0.66–1.75)	0.76	1.45 (0.82–2.56)	0.19
Alcohol consumption				
Abstainers	1		1	
Drinker or former‐drinker	1.54 (0.96–2.45)	0.07	1.02 (0.60–1.73)	0.93
Clinical stage				
Early (I + II)	1		1	
Advanced (III + IV)	1.76 (1.04–2.98)	0.03	1.85 (1.00–3.45)	0.05
Tumor site				
Tongue	1		1	
Floor of mouth	1.23 (0.96–1.57)	0.09	1.07 (0.77–1.48)	0.68
Others	1.32 (0.95–1.82)	0.08	1.14 (0.76–1.71)	0.52
Treatment				
Surgery	1		1	
Surgery + Radiotherapy	1.33 (0.81–2.20)	0.26	0.73 (0.42–1.29)	0.28
Surgery + Radiotherapy + Chemotherapy	0.97 (0.59–1.58)	0.90	0.89 (0.56–1.40)	0.63
Margin status				
≥ 5 mm	1		1	
< 5 mm	0.97 (0.48–1.96)	0.94	1.20 (0.60–2.39)	0.59
WHO histopathological grading				
Well‐differentiated	1		1	
Moderately differentiated	0.76 (0.47–1.25)	0.28	0.90 (0.51–1.57)	0.71
Poorly differentiated	0.99 (0.66–1.50)	0.99	0.92 (0.57–1.50)	0.76
Perineural invasion (PNI)				
No	1		1	
Yes	2.26 (1.43–3.59)	0.0005	1.45 (0.84–2.51)	0.17
Lymphovascular invasion (LVI)				
No	1		1	
Yes	2.00 (1.21–3.31)	0.006	1.14 (0.59–2.22)	0.69
Worst pattern of invasion (WPOI)				
Pattern 1 + 2 + 3	1		1	
Pattern 4 + 5	1.95 (1.23–3.09)	0.004	2.06 (1.21–3.51)	0.007
Tumor budding (TB)				
< 5 buds	1		1	
≥ 5 buds	1.82 (1.14–2.91)	0.01	2.17 (1.26–3.73)	0.005
Modified WPOI				
0	1		1	
1	1.05 (0.33–3.35)	0.93	1.25 (0.30–5.14)	0.09
2	1.13 (0.50–2.53)	0.76	1.88 (0.53–6.67)	0.32
Modified TB				
0	1		1	
1	0.98 (0.49–2.00)	0.97	1.15 (0.84–1.57)	0.38
2	1.21 (0.91–1.63)	0.18	1.49 (0.64–3.44)	0.36
Histopathologic risk model				
Low	1		1	
Intermediate	1.26 (0.73–2.19)	0.39	1.00 (0.58–1.73)	0.97
High	1.31 (0.62–2.75)	0.47	1.18 (0.64–2.17)	0.58

**TABLE 2:** Multivariate analysis of cancer‐specific survival and disease‐specific survival for the patients with oral squamous cell carcinoma.

	Cancer‐specific survival		Disease‐specific survival	
	HR (95% CI)	*p*	HR (95% CI)	*p*
Model 1				
Worst pattern of invasion (WPOI)	2.02 (1.26–3.25)	0.003		
Perineural invasion (PNI)	1.68 (1.05–2.70)	0.02		
Tumor budding (TB)			2.17 (1.25–3.74)	0.005
Model 2				
Perineural invasion (PNI)	2.26 (1.42–3.58)	0.005		
Model 3				
Perineural invasion (PNI)	2.26 (1.42–3.58)	0.005		

*Note*: Model 1: Clinicopathological variables and worst pattern of invasion and tumor budding. Model 2: Clinicopathological variables and modified worst pattern of invasion and modified tumor budding. Model 3: Clinicopathological variables and histopathologic risk model.

Survival analyses of the histopathological risk model revealed no significant associations with both cancer‐specific survival and disease‐free survival (Table 1). When modified WPOI and modified TB, or the histopathological risk model, were incorporated into the Cox models, perineural invasion (PNI) emerged as the only independent risk factor for cancer‐specific survival (Table 2).

## 4 Discussion

Although efforts to standardize and simplify histopathological assessment systems, such as the modification on WPOI and TB proposed by Chang et al. [9], are commendable for reducing interobserver variability and minimizing overlapping criteria, the findings of the current study highlight some limitations. The significant prognostic differences between WPOI‐3 and WPOI‐4 (Data S4) emphasize the importance of maintaining these two categories. Grouping them results in most OSCC cases falling within pattern 1 (invasive tumor islands of any size), with only a few cases categorized under pattern 0, traditionally related to tumors at the initial stage of invasion, or under pattern 2 (tumor satellite). Another critical concern is the proposed modifications to TB classification. Most OSCCs at advanced stages display one or other TB, but not enough to surpass the threshold for high TB activity (≥ 5 buds), which supports the appropriateness of this cutoff. Additionally, tumors with isolated cells frequently show high TB activity, leading to consistently elevated scores in both the classical and modified classification systems. This substantial overlap diminishes the incremental value of the modifications proposed by Chang et al. [9], as incorporating single cells into the TB score does not appear to significantly enhance prognostic stratification. Furthermore, the restrictive definition of high‐risk cases in the histological risk model, which requires presence of tumor satellite, is relatively uncommon in OSCC, as found in the current study and others [12], limits its performance. Only 3.1% of the cases in the current cohort were classified as high‐risk, whereas the vast majority (68.4%) fell into the intermediate‐risk category.

In summary, the findings of this study indicate that classical WPOI and classical BD have superior prognostic value compared to modified systems. Additionally, the evaluation of the novel histopathological risk model did not prove effective in predicting patient outcomes across multiple clinical stages of OSCC.

## Author Contributions

**Everton Freitas de Morais:** conceptualization, data curation, formal analysis, investigation, methodology, writing – original draft, writing – review and editing. **Lívia Maris Ribeiro Paranaiba Dias:** data curation, formal analysis, funding acquisition, writing – review and editing. **Ana Lúcia Carrinho Ayroza Rangel:** data curation, formal analysis, writing – review and editing. **Ricardo D. Coletta:** conceptualization, data curation, formal analysis, funding acquisition, investigation, methodology, project administration, supervision, writing – original draft, writing – review and editing.

## Ethics Statement

This study was conducted in accordance with the Declaration of Helsinki. This study was approved by the Human Research Ethics Committee of the School of Dentistry, University of Campinas (CAAE: 55927322.0.0000.5418).

## Conflicts of Interest

The authors declare no conflicts of interest.

## Peer Review

The peer review history for this article is available at https://www.webofscience.com/api/gateway/wos/peer‐review/10.1111/jop.70046.

## Supporting information


**Data S1:** Clinicopathological features of 193 patients with oral squamous cell carcinoma included in this study.

**Data S2:** Schematic representation of classical and modified worst pattern of invasion (WPOI) and tumor budding (TB), and the novel histopathological risk model. (A) The classical WPOI categorizes invasion into 5 tiers (WPOI‐1 to WPOI‐5), whereas the modified WPOI simplifies it within three tiers (score 0 to score 2). Briefly, score 0 corresponds to WPOI‐1 and WPOI‐2, score 1 includes WPOI‐3 and WPOI‐4, and score 2 represents WPOI‐5. (B) The classical TB employs a cut off, dividing the tumors into either low budding (0–4 buds) or high budding (≥ 5 buds). The modified TB utilizes three levels: score 0 indicates complete absence of buds, score 1 included 1–4 buds, and score 2 corresponds to 5 or more buds or presence of single tumor cells. (C) The novel histopathologic risk model based on combination of the modified WPOI and modified TB.

**Data S3:** Distribution of 193 cases of oral squamous cell carcinoma according to the classical and modified classification of the worst pattern of invasion (WPOI) and tumor budding (TB). The histopathologic risk model was determined based on the modified WPOI and TB, following the system proposed by Chang et al. (2024).

**Data S4:** Kaplan–Meier survival curves for classical WPOI‐3 and WPOI‐4. (A) Disease‐free survival, and (B) cancer‐specific survival.

## Data Availability

The data to support the findings of this study will be available on request from the corresponding author.
