# Supplementary material for: Prognostic Significance of a Novel Histopathologic Risk Model Incorporating Modifications to the Worst Pattern of Invasion and Tumor Budding for Oral Squamous Cell Carcinoma
Source: J Oral Pathol Med. 2025 Aug 19;54(9):903–8. doi: 10.1111/jop.70046 (PMC12521064; doi:10.1111/jop.70046)
Supplement: Supplementary file 1 — Data S1: Clinicopathological features of 193 patients with oral squamous cell carcinoma included in this study. [file JOP-54-903-s004.docx]

**Supplementary File 1.** Clinicopathological features of 193 patients with oral squamous cell carcinoma included in this study.

|  | n (%) |
| --- | --- |
| Age (years) |  |
| Mean ± Standard derivation | 58.3 ± 10.9 |
| Range | 17-88 |
| Sex |  |
| Male | 150 (77.7) |
| Female | 43 (22.3) |
| Smoking |  |
| Never-smoker | 64 (33.2) |
| Smoker or Former-smoker | 100 (51.8) |
| Missing | 29 (15.0) |
| Alcohol consumption |  |
| Abstainers | 52 (26.9) |
| Drinker or Former-drinker | 99 (51.3) |
| Missing | 42 (21.8) |
| Clinical stage |  |
| I | 39 (20.2) |
| II | 54 (28.0) |
| III | 56 (20.7) |
| IV | 60 (31.1) |
| Location |  |
| Tongue | 128 (66.4) |
| Floor of mouth | 46 (23.8) |
| Retromolar area | 15 (7.8) |
| Palate | 2 (1.0) |
| Gingiva | 2 (1.0) |
| Treatment |  |
| Surgery | 67 (34.7) |
| Surgery + Radiotherapy | 105 (54.4) |
| Surgery + Radiotherapy + Chemotherapy | 21 (10.9) |
| Margin status |  |
| ≥5 mm | 161 (83.4) |
| <5 mm | 32 (16.6) |
| WHO histopathological grading |  |
| Well-differentiated | 77 (39.9) |
| Moderately-differentiated | 97 (50.3) |
| Poorly-differentiated | 19 (9.8) |
| Perineural invasion |  |
| No | 127 (65.8) |
| Yes | 66 (32.2) |
| Lymphovascular invasion |  |
| No | 151 (78.2) |
| Yes | 42 (21.8) |
| Local recurrence |  |
| No | 158 (81.9) |
| Yes | 35 (18.1) |
| Regional recurrence |  |
| No | 179 (92.7) |
| Yes | 14 (7.3) |
| Distant recurrence |  |
| No | 182 (94.3) |
| Yes | 11 (5.7) |
| Status |  |
| Alive | 120 (62.2) |
| Dead | 73 (37.8) |
