# Supplementary figures and images for: Prognostic Significance of a Novel Histopathologic Risk Model Incorporating Modifications to the Worst Pattern of Invasion and Tumor Budding for Oral Squamous Cell Carcinoma
Source: J Oral Pathol Med. 2025 Aug 19;54(9):903–8. doi: 10.1111/jop.70046 (PMC12521064; doi:10.1111/jop.70046)

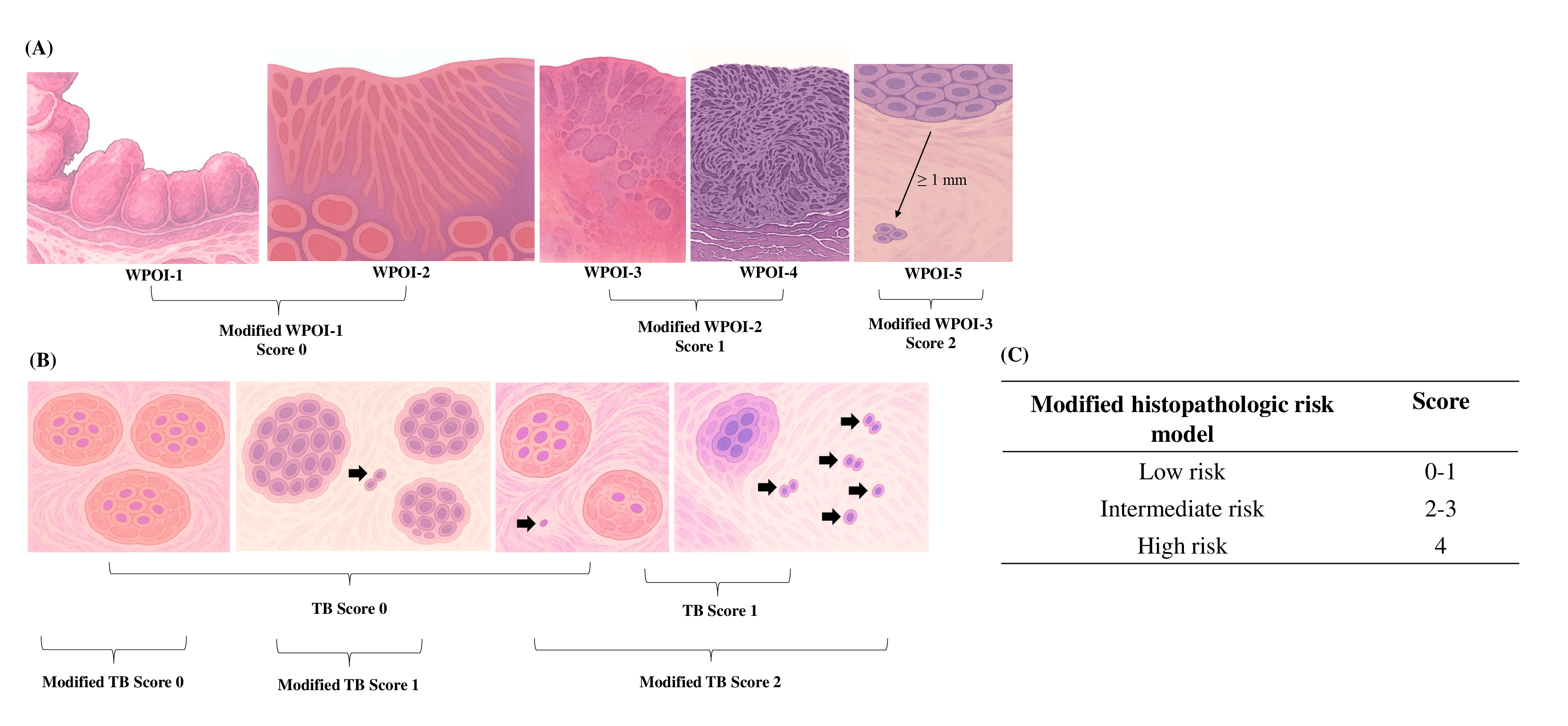

Supplement: Supplementary file 2 — Data S2: Schematic representation of classical and modified worst pattern of invasion (WPOI) and tumor budding (TB), and the novel histopathological risk model. (A) The classical WPOI categorizes invasion into 5 tiers (WPOI‐1 to WPOI‐5), whereas the modified WPOI simplifies it within three tiers (score 0 to score 2). Briefly, score 0 corresponds to WPOI‐1 and WPOI‐2, score 1 includes WPOI‐3 and WPOI‐4, and score 2 represents WPOI‐5. (B) The classical TB employs a cut off, dividing the tumors into either low budding (0–4 buds) or high budding (≥ 5 buds). The modified TB utilizes three levels: score 0 indicates complete absence of buds, score 1 included 1–4 buds, and score 2 corresponds to 5 or more buds or presence of single tumor cells. (C) The novel histopathologic risk model based on combination of the modified WPOI and modified TB. [file JOP-54-903-s002.png]

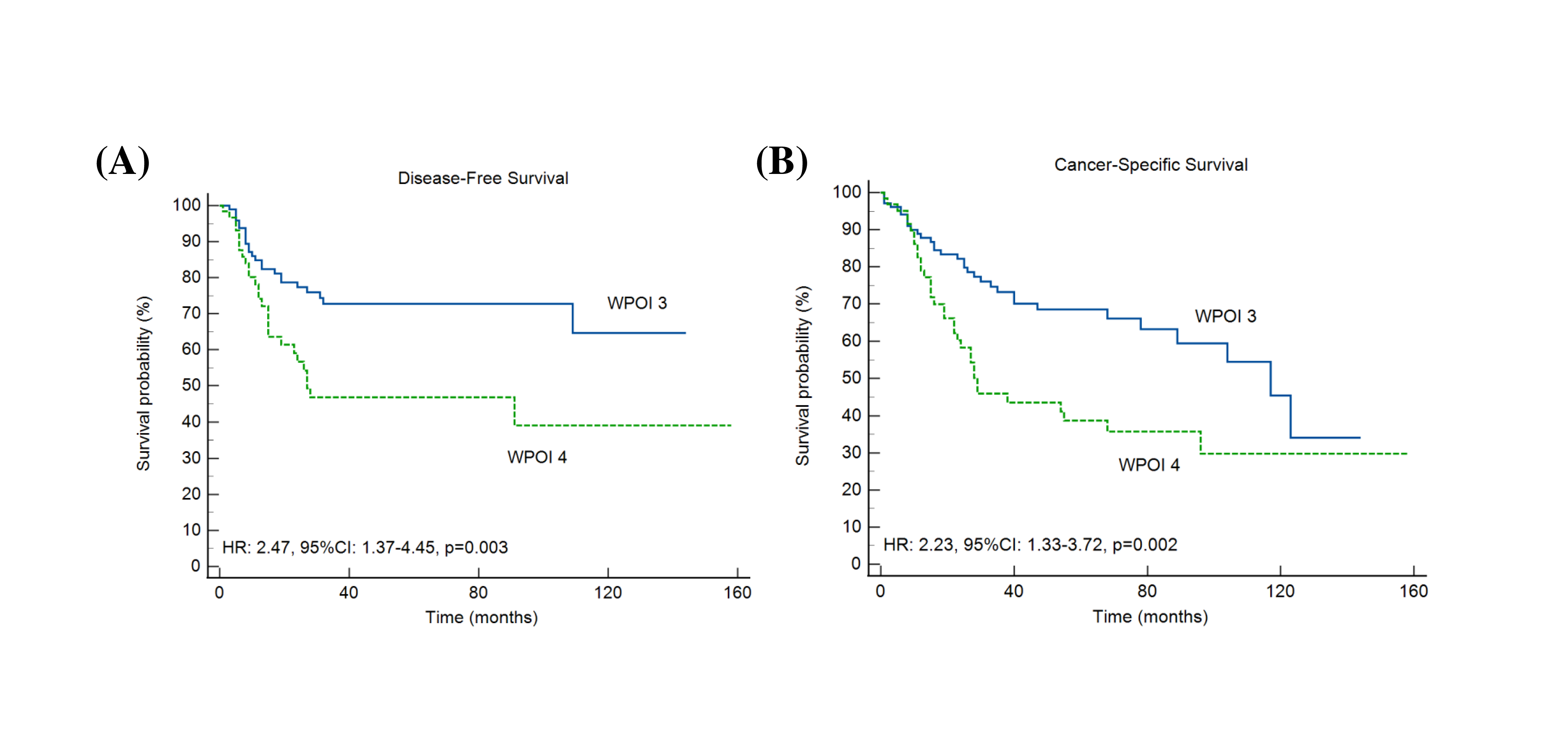

Supplement: Supplementary file 4 — Data S4: Kaplan–Meier survival curves for classical WPOI‐3 and WPOI‐4. (A) Disease‐free survival, and (B) cancer‐specific survival. [file JOP-54-903-s001.tiff]
