# Supplementary material for: Prognostic Significance of a Novel Histopathologic Risk Model Incorporating Modifications to the Worst Pattern of Invasion and Tumor Budding for Oral Squamous Cell Carcinoma
Source: J Oral Pathol Med. 2025 Aug 19;54(9):903–8. doi: 10.1111/jop.70046 (PMC12521064; doi:10.1111/jop.70046)
Supplement: Supplementary file 3 — Data S3: Distribution of 193 cases of oral squamous cell carcinoma according to the classical and modified classification of the worst pattern of invasion (WPOI) and tumor budding (TB). The histopathologic risk model was determined based on the modified WPOI and TB, following the system proposed by Chang et al. (2024). [file JOP-54-903-s003.docx]

**Supplementary File 3.** Distribution of 193 cases of oral squamous cell carcinoma according to the classical and modified classification of the worst pattern of invasion (WPOI) and tumor budding (TB). The histopathologic risk model was determined based on the modified WPOI and TB, following the system proposed by Chang et al. (2024).

| Parameter | n (%) |
| --- | --- |
| Worst pattern of invasion (WPOI) |  |
| 1 (Pushing border) | 5 (2.6) |
| 2 (Finger-like growth) | 10 (5.2) |
| 3 (Large tumor islands) | 104 (53.9) |
| 4 (Small tumor islands) | 64 (33.1) |
| 5 (Tumor satellite) | 10 (5.2) |
| Tumor budding (TB) |  |
| < 5 buds | 101 (52.3) |
| ≥ 5 buds | 92 (47.7) |
| Modified WPOI |  |
| 0 (Pushing border) | 7 (3.6) |
| 1 (Tumor islands of any size) | 176 (91.2) |
| 2 (Tumor satellite) | 10 (5.2) |
| Modified TB |  |
| 0 (No buds) | 53 (27.5) |
| 1 (1 to 4 buds) | 43 (22.3) |
| 2 (≥ 5 buds or presence of isolated cells) | 97 (50.3) |
| Histopathologic risk model |  |
| Low | 55 (28.5) |
| Intermediate | 132 (68.4) |
| High | 6 (3.1) |
